# Identification of Pan-Cancer Biomarkers Based on the Gene Expression Profiles of Cancer Cell Lines

**DOI:** 10.3389/fcell.2021.781285

**Published:** 2021-11-30

**Authors:** ShiJian Ding, Hao Li, Yu-Hang Zhang, XianChao Zhou, KaiYan Feng, ZhanDong Li, Lei Chen, Tao Huang, Yu-Dong Cai

**Affiliations:** ^1^ School of Life Sciences, Shanghai University, Shanghai, China; ^2^ College of Food Engineering, Jilin Engineering Normal University, Changchun, China; ^3^ Channing Division of Network Medicine, Brigham and Women’s Hospital, Harvard Medical School, Boston, MA, United States; ^4^ Center for Single-Cell Omics, School of Public Health, Shanghai Jiao Tong University School of Medicine, Shanghai, China; ^5^ Department of Computer Science, Guangdong AIB Polytechnic College, Guangzhou, China; ^6^ College of Information Engineering, Shanghai Maritime University, Shanghai, China; ^7^ CAS Key Laboratory of Computational Biology, Bio-Med Big Data Center, Shanghai Institute of Nutrition and Health, University of Chinese Academy of Sciences, Chinese Academy of Sciences, Shanghai, China; ^8^ CAS Key Laboratory of Tissue Microenvironment and Tumor, Shanghai Institute of Nutrition and Health, University of Chinese Academy of Sciences, Chinese Academy of Sciences, Shanghai, China

**Keywords:** pan-cancer study, feature selection, classification algorithm, decision rule, biomarker

## Abstract

There are many types of cancers. Although they share some hallmarks, such as proliferation and metastasis, they are still very different from many perspectives. They grow on different organ or tissues. Does each cancer have a unique gene expression pattern that makes it different from other cancer types? After the Cancer Genome Atlas (TCGA) project, there are more and more pan-cancer studies. Researchers want to get robust gene expression signature from pan-cancer patients. But there is large variance in cancer patients due to heterogeneity. To get robust results, the sample size will be too large to recruit. In this study, we tried another approach to get robust pan-cancer biomarkers by using the cell line data to reduce the variance. We applied several advanced computational methods to analyze the Cancer Cell Line Encyclopedia (CCLE) gene expression profiles which included 988 cell lines from 20 cancer types. Two feature selection methods, including Boruta, and max-relevance and min-redundancy methods, were applied to the cell line gene expression data one by one, generating a feature list. Such list was fed into incremental feature selection method, incorporating one classification algorithm, to extract biomarkers, construct optimal classifiers and decision rules. The optimal classifiers provided good performance, which can be useful tools to identify cell lines from different cancer types, whereas the biomarkers (e.g. NCKAP1, TNFRSF12A, LAMB2, FKBP9, PFN2, TOM1L1) and rules identified in this work may provide a meaningful and precise reference for differentiating multiple types of cancer and contribute to the personalized treatment of tumors.

## Introduction

“Cancer” is the term used to describe a series of diseases that is characterized by the spontaneous expansion and spread of somatic cell clones. It is becoming a serious public health problem worldwide. In 2020 alone, over 19.29 million new cases of cancer were diagnosed, and more than 9.58 million people died from cancer (World Health Organization, 2019). The hallmarks of cancer have been extensively described as six biological capabilities, namely, enhanced proliferative signaling, growth suppressor escape, cell death resistance, replicative immortality, angiogenesis induction, and invasion and metastasis activation (Hanahan and Weinberg, 2011). In other words, the pro-oncogenic function is the abnormal expression of various genes based on these six biological capabilities. Therefore, cancer genomic data, particularly gene expression signatures, can provide insight into the occurrence and development of cancers and, importantly, can be used to develop targeted therapies for cancers (Garman et al., 2007).

Although many cancers share the hallmarks of cancer, they are still very different. They grow on different organs and tissue. Pan-cancer studies provide an opportunity to understand the commonalities, heterogeneity, and emergent themes of multiple tumors (Andor et al., 2016). Increased numbers of tumor sample datasets provide scientists with a clear picture of tumors, rare driver events in heterogeneous tumor samples, and new molecular carcinogenic mechanisms that may be readily detected (Weinstein et al., 2013). For example, a study on the genomic predictors of the drug sensitivity of 947 human cancer cell lines based on a cancer cell line encyclopedia revealed known and novel response candidate biomarkers, which may contribute to cancer biology and therapeutic development (Barretina et al., 2012). Another study on long noncoding RNA (lncRNA) in 5185 TCGA tumors demonstrated that although tumor-specific dysregulated lncRNAs are commonly observed in a variety of tumors, genes and pathways could be synergistically regulated in different cancers by the same group of lncRNAs; this information may provide useful ideas for the development of broad-spectrum antineoplastic drugs (Chiu et al., 2018).

The sample size of TCGA based pan-cancer studies is already very large as a multi-omics data source. But it is still not enough to get robust pan-cancer biomarkers if we consider the large variances among cancer patients across cancer types and even within the same cancer. Tumor heterogeneity can be broadly categorized into intratumor heterogeneity and inter tumor heterogeneity (Burrell and Swanton, 2014). Inter tumor heterogeneity refers to the heterogeneity between patients with the same histological tumor type and has been considered to be caused by patient-specific factors, including germline mutations, individualized somatic mutations, and environmental factors. Intratumor heterogeneity can be divided into spatial heterogeneity (different regions of the tumor have different genetic aberrations) and temporal heterogeneity (during disease progression) (Dagogo-Jack and Shaw, 2018). Studies across multiple cancers have suggested that intratumor heterogeneity promotes tumor growth, metastasis, and drug resistance in human cancers (Hyo-eun et al., 2015; Russo et al., 2016). Therefore, treatment strategies with increased effectiveness and durability still need to be developed on the basis of a comprehensive understanding of tumor dynamics.

To get robust pan-biomarkers, there are two approaches: increase the sample size or reduce the variance. TCGA and the following works tried the first approach of increasing sample size. In this study, we would like to try the second approach of reducing the variance by analyzing the cancer cell line data from Cancer Cell Line Encyclopedia (CCLE) (Ghandi et al., 2019). The important genes were extracted by using the Boruta method (Kursa and Rudnicki, 2010). These genes were further analyzed with the max-relevance and min-redundancy (mRMR) method to evaluate their importance and sort them in a feature list. This list was fed into the incremental feature selection (IFS) method (Liu and Setiono, 1998) that combined support vector machine (SVM) (Cortes and Vapnik, 1995) or decision tree (DT) (Safavian and Landgrebe, 1991) to identify important genes and decision rules and build powerful classifiers. Further analysis was performed through a literature review of the top-ranked genes and portion decision rules to confirm the validity and reliability of the results. This study gives new insight into pan-cancer studies and may provide novel targets of tumor-specific therapies.

## Materials and Methods

### Datasets

Xiao et al. (Xiao et al., 2019) downloaded the raw RNA-Seq data from Cancer Cell Line Encyclopedia (CCLE) (Ghandi et al., 2019) and quantified the gene expression levels as Transcripts Per kilobase Million (TPM) using RSEM (Li and Dewey, 2011). We used the processed gene expression data by Xiao et al. (Xiao et al., 2019). The cancer types with sample sizes of less than 10 was removed. Finally, there were 988 cancer cell lines from 20 cancer types. The sample size of each tumor is listed in Table 1. For each sample, 57,820 gene features were included. We investigated the expression patterns of genes in different tumor types and whether these tumor types could be distinguished on the basis of expression profiles.

**TABLE 1:** Distribution of samples and decision rules in different cancer cell lines.

Cancer cell line types	Number of cell lines	Number of decision rules	Number of criteria	Number of involved genes
Autonomic ganglia	16	4	42	17
Bone	20	4	46	19
Breast	51	23	325	63
Central nervous system	65	18	219	57
Endometrium	28	16	191	52
Fibroblast	37	3	28	15
Haematopoietic and lymphoid tissue	173	8	96	34
Kidney	32	7	88	38
Large intestine	56	9	123	47
Liver	25	9	115	42
Lung	188	51	740	80
Oesophagus	27	8	145	47
Ovary	47	25	306	67
Pancreas	41	14	208	56
Skin	49	7	83	34
Soft tissue	28	9	126	41
Stomach	37	26	361	72
Thyroid	12	7	79	38
Upper aerodigestive tract	31	11	162	47
Urinary tract	25	16	216	59

### Boruta Feature Selection

The CCLE data involved a large number of genes (features). Obviously, not all genes are associated with the investigated tumor types. Therefore, filtering the important genes is necessary. Here, we applied the Boruta (Kursa and Rudnicki, 2010) method to select a set of relevant features with multiple tumor labels.

The Boruta method is a wrapping algorithm that is based on random forest (RF) and involves the following steps: 1) the new shuffled data are generated by copying the original dataset and shuffling original features; 2) a RF classifier that can output the importance score of each feature is trained by using the new feature matrix as the input; and 3) the features in the original features that are sincerely relevant to the labels are retained, and the shuffled data are removed. Boruta finally selects the relevant features after several iterations of the above three steps.

The Boruta program that we used in this research was downloaded from https://github.com/scikit-learn-contrib/boruta_py and was set to default parameters for execution.

### mRMR

After feature filtering by using the Boruta method, the mRMR (Peng et al., 2005) feature selection method was used to evaluate the importance of the remaining features. This approach has been widely used to analyze complicated systems.

The mRMR method evaluates the importance of target features by using max-relevance and min-redundancy. Features with great relevance to the category labels and low redundancy with other features are considered to be influential. It uses mutual information (MI) to measure relevance and redundancy. The score of the MI between two variables  X and Y  is calculated as

$$I\left(X,Y\right)=\int \int p\left(x,y\right)\log\frac{p\left(x,y\right)}{p\left(x\right)p\left(y\right)}\mathrm{dxdy},$$ (1)

where p(x,y) represents the joint probability distribution function of X and Y , and p(x) and  p(y) represent the marginal probability distribution function of X and Y , respectively. The mRMR constructs order feature lists on the basis of the importance of features. Specifically, the program loops several times, and each loop selects a feature that has the greatest correlation with the target variable and the least correlation with the selected features. Finally, a list of sorting features is obtained in accordance with the selected orders.

The mRMR program used in this research was obtained from http://penglab.janelia.org/proj/mRMR/and executed with default parameters.

### Incremental Feature Selection

Even though the mRMR method produces a ranked list of features on the basis of the importance of features, we still cannot determine the influential features. The IFS (Liu and Setiono, 1998) method can determine the optimal number of key features in combination with one classification algorithm. First, IFS generates a series of feature subsets from the above feature list in accordance with the step size. For example, if the step size is 10, the first feature subset will be the top 10 features, and the second subset will be the top 20 features. Next, one classifier is built based on the training set, where the samples are represented by features from each feature subset. The classifiers are evaluated by 10-fold cross-validation (Kohavi, 1995) to obtain evaluation metrics. Finally, the optimal feature subset and the best classifier are determined, and features in this subset are called optimum features.

### Decision Tree

DT (Safavian and Landgrebe, 1991) is a model that presents decision rules and classification results in a tree-like structure and is widely used in the biological and biomedical fields. DT is a supervised learning approach that builds a model based on the IF–THEN format. It achieves superior model performance through low computational complexity. The common decision trees are Iterative Dichotomizer 3, C4.5, and Classification and Regression Tree. They use different partition strategies when building a prediction model. In this study, we used the Scikit-learn (Pedregosa et al., 2011) module in Python to construct a DT classifier.

### Support Vector Machine

SVM (Cortes and Vapnik, 1995; Zhou J.-P. et al., 2020; Wang et al., 2021) is a supervised learning algorithm in statistical learning methods that is commonly used in classification and regression problems. SVM maps the data from a low-dimensional space to a high-dimensional space by using a kernel function. Then, a hyperplane with the maximum interval existing in the high-dimensional space makes two classes of samples linearly separable.

In this study, 20 tumor types needed to be classified. This task was a multiclass classification problem. Therefore, we applied the one-versus-rest strategy to train a multiclass SVM, which was split into numerous binary SVMs. For each binary SVM, samples of one class were regarded as positive examples, and samples of all other classes were used as negative examples. We directly used the tool “SMO” in Weka software (Gewehr et al., 2007) in this study. The sequential minimum optimization algorithm (Platt, 1998; Keerthi et al., 2001) was utilized to optimize the training procedure. The kernel function was set as a polynomial function.

### Synthetic Minority Oversampling Technique

As can be seen from Table 1, the sample sizes for all tumor types were quite different. For example, types “lung” and “hematopoietic and lymphoid tissue” contained 188 and 173 samples, respectively, whereas types “autonomic ganglion” and “bone” had only 16 and 20 samples, respectively. These results indicated that the whole dataset of this study was unbalanced. Accordingly, we adopted the synthetic minority oversampling technique (SMOTE) (Chawla et al., 2002) to balance the dataset when building classifiers. This method uses the k-nearest neighbor algorithm to expand the sample sizes of each minority class. SMOTE first selects random data from one minority class and then finds the k-nearest neighbors in this class. Next, the new sample data are synthesized between the random data and the randomly generated k-nearest neighbor. After SMOTE processing, the sample size of each minority class is equal to that of the majority class. In other words, the sample sizes of the 20 tumor types in this study were equal. In this study, we oversampled data by using the tool “SMOTE” in Weka software (Gewehr et al., 2007).

### Performance Measurement

In this study, several multiclass classifiers were used to distinguish samples from 20 tumor types. We adopted 10-fold cross-validation (Kohavi, 1995; Chen et al., 2017; Zhao et al., 2018; Zhou JP. et al., 2020; Jia et al., 2020; Liang et al., 2020; Zhang et al., 2021c; Yang and Chen, 2021; Zhu et al., 2021) to evaluate the performance of each multiclass classifier. We correlation coefficient (MCC) (Matthews, 1975; Gorodkin, 2004; Liu H. et al., 2021; Zhang et al., 2021a; Zhang et al., 2021b; Pan et al., 2021) to measure and evaluate the prediction quality of the results of 10-fold cross-validation. Let *X* be a matrix representing predicted labels yielded by one classifier and *Y* be another matrix indicating the actual labels of samples. The calculation formula of MCC is as follows:

$$MCC=\;\frac{cov\left(X,Y\right)}{\sqrt{cov\left(X,X\right)cov\left(Y,Y\right)}}=\;\frac{\sum_{i=1}^{n}\sum_{j=1}^{C}\left(x_{ij}-{\bar{x}}_{j}\right)\left(y_{ij}-{\bar{y}}_{j}\right)}{\sqrt{\sum_{i=1}^{n}\sum_{j=1}^{C}{\left(x_{ij}-{\bar{x}}_{j}\right)}^{2}\sum_{i=1}^{n}\sum_{j=1}^{C}{\left(y_{ij}-{\bar{y}}_{j}\right)}^{2}}},$$ (2)

where cov(X,Y) denotes the correlation coefficient of X and Y , and ${\bar{x}}_{j}$ and ${\bar{y}}_{j}\;$ are the average values in the j th column of X and Y , respectively. In addition, C  denotes the number of tumor types, and n denotes the total number of samples.

In addition to MCC, we also calculated accuracy on each cancer type and overall accuracy. The accuracy on the *i*th cancer type was computed by

$$Accuracy_{i}=\frac{n_{i}}{N_{i}}\;\;\;\;\;\;\;\;\;\;\text{i}=1,2,\cdots ,20,$$ (3)

where *N*_*i*_ represents the number of samples in the *i*th cancer type and *n*_*i*_ denotes correctly predicted samples in the *i*th cancer type. The overall accuracy was calculated by

$$\text{Overall accuracy}=\frac{\sum_{i=1}^{20}n_{i}}{\sum_{i=1}^{20}N_{i}}\;\;\;\;\;\;\;\;\;\text{ i}=1,2,\cdots ,20,$$ (4)

MCC was set as the key measurement and others were also provided for reference in this study.

## Results

**FIGURE 1:**
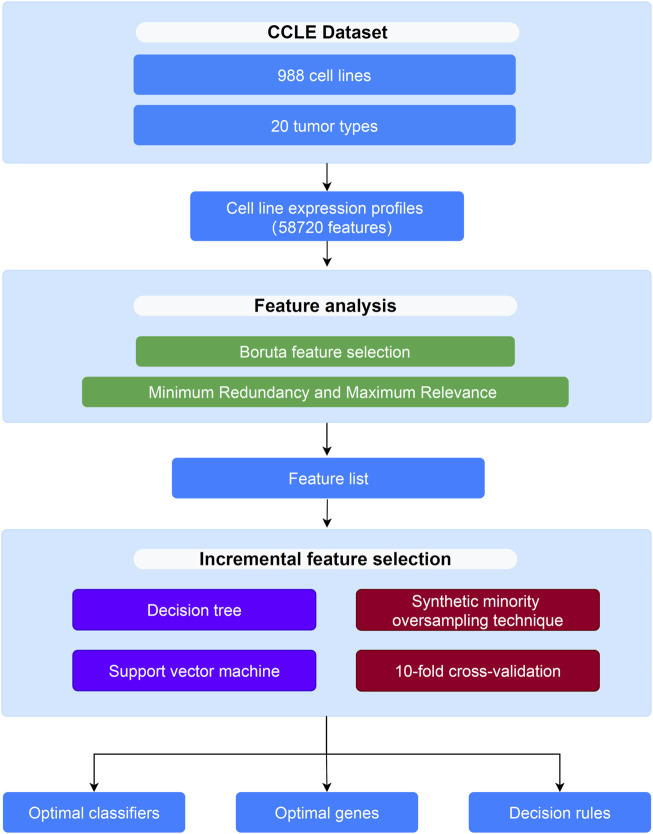
Flow chart to show the entire analysis procedures. The CCLE dataset which includes 988 cell lines and 20 tumor types is analyzed by Boruta and mRMR methods, resulting in a feature list. The list is then fed into the incremental feature selection method to extract optimal genes, build the optimal classifiers and construct decision rules.

In this study, several computational methods were used to analyze the CCLE dataset of 20 tumor types. The analysis process is shown in Figure 1.

### Results of the Boruta and max-Relevance and Min-Redundancy Methods

All features were first analyzed by using the Boruta method. A total of 54,634 features were removed, and 3,186 features were retained. These retained features are provided in Supplementary Table S1. These 3,186 features were further analyzed by using the mRMR method, and a feature ranking list was generated on the basis of their importance. This list can also be found in Supplementary Table S1.

### Results of the Incremental Feature Selection Method

**FIGURE 2:**
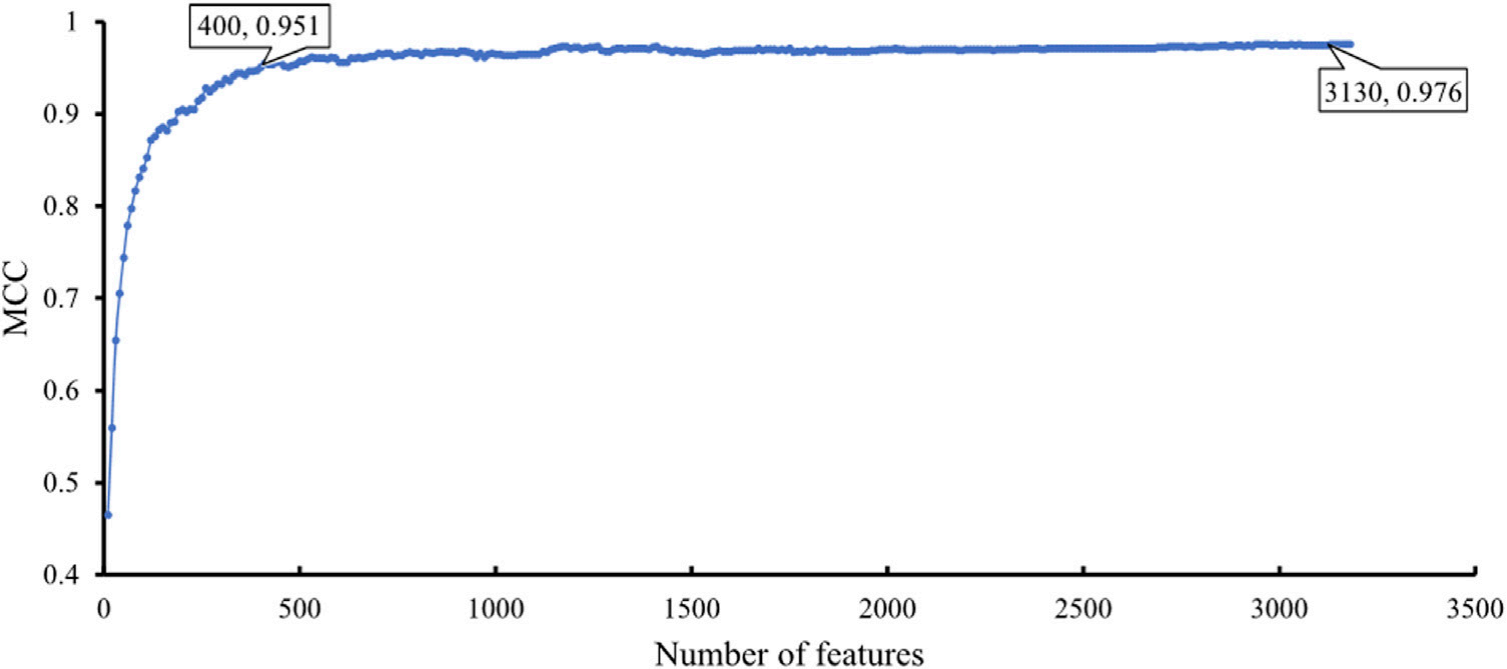
IFS curve with SVM classification algorithm on the different number of features. The SVM provides the highest MCC of 0.976 when the top 3,130 features are adopted. When top 400 features are adopted, SVM provides good performance with MCC of 0.951.

**FIGURE 3:**
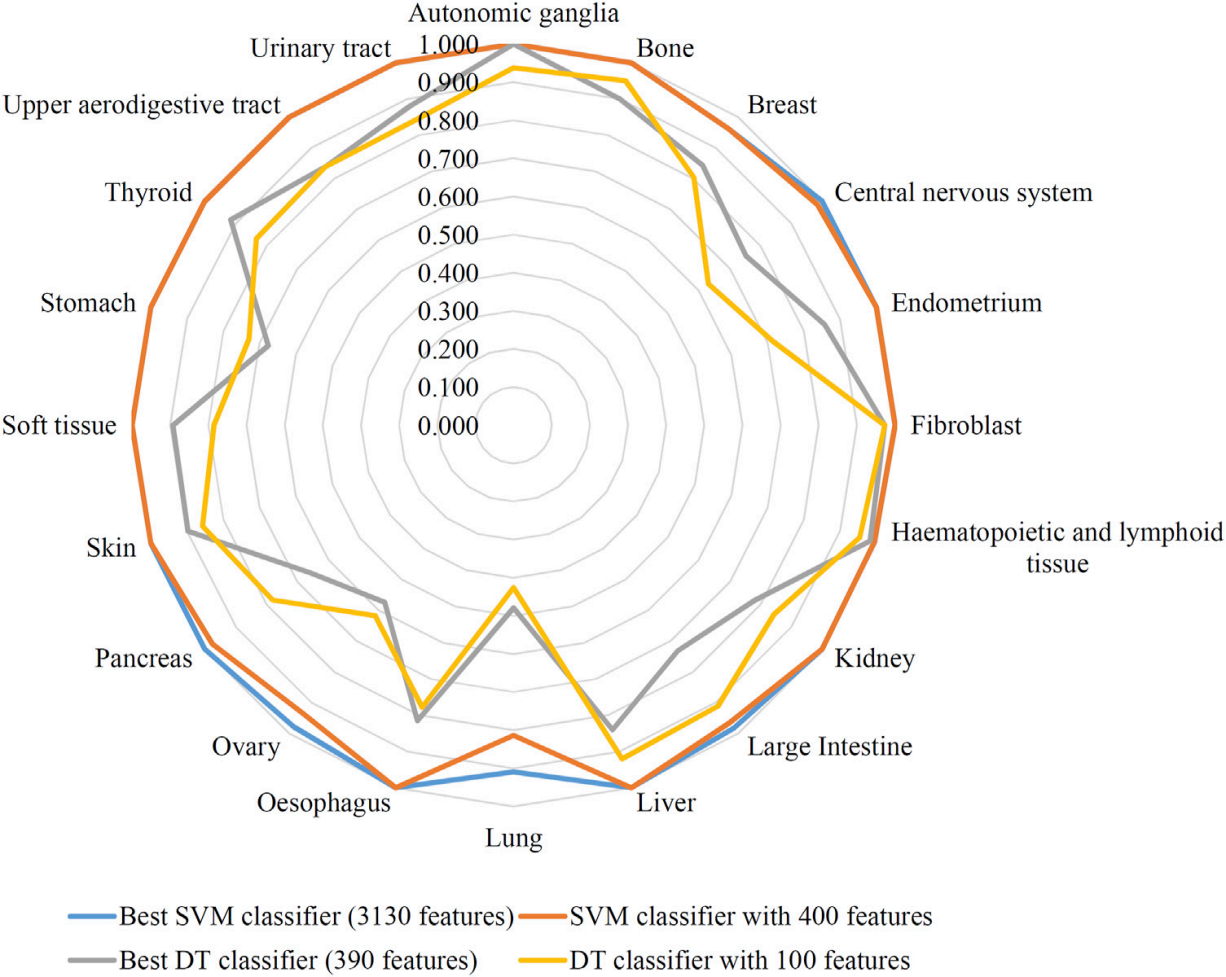
Radar graph to show the performance of two support vector machine (SVM) classifiers and two decision tree (DT) classifiers on 20 cancer types. Two SVM classifiers provide almost equal performance, also for two DT classifiers.

The feature list produced by the mRMR method was fed into the IFS method. A series of feature subsets were generated by setting the step size to 10. The DT and SVM were used to build classifiers on each feature subset. Then, all classifiers were evaluated by 10-fold cross-validation to obtain evaluation metrics, such as accuracy on each cancer type, overall accuracy and MCC. The above measurements that were acquired by the two classification algorithms for each subset of features are shown in Supplementary Table S2. We plotted the IFS curve to visualize the results. For SVM, the MCC was set as the Y-axis, and the number of features was set as the X-axis. As shown in Figure 2, when the number of features reached 3,130, the highest value of MCC was 0.976. The corresponding overall accuracy was 0.978 (Table 2). Accordingly, the best SVM classifier can be built based on these top 3,130 features. Although this classifier provided the highest performance, its efficiency was not very high because an excessively high number of features were used. The IFS results of SVM were carefully checked (Supplementary Table S2 and Figure 2). When the top 400 features were adopted, the MCC reached 0.951, which was only slightly lower than the highest MCC. The overall accuracy was 0.954 (Table 2). It was also a little lower than that of the best SVM classifier. It can be concluded that these two SVM classifiers provided almost equal performance. To further confirm this fact, we also investigated the accuracies on 20 cancer types yielded by these two classifiers. A radar graph was plotted, as shown in Figure 3. Clearly, the areas inside the curves of two classifiers were almost same, suggesting the equal performance of these two classifiers. However, the number of features was considerably lower. The SVM classifier with these features had drastically higher efficiency than the best SVM classifier. Thus, this classifier could be the proposed classifier for assigning samples to the correct cancer type.

**TABLE 2:** Performance of some key classifiers.

Classification algorithm	Number of features	Overall accuracy	MCC
Support vector machine	3,130	0.978	0.976
	400	0.954	0.951
Decision tree	390	0.771	0.754
	100	0.757	0.739

**FIGURE 4:**
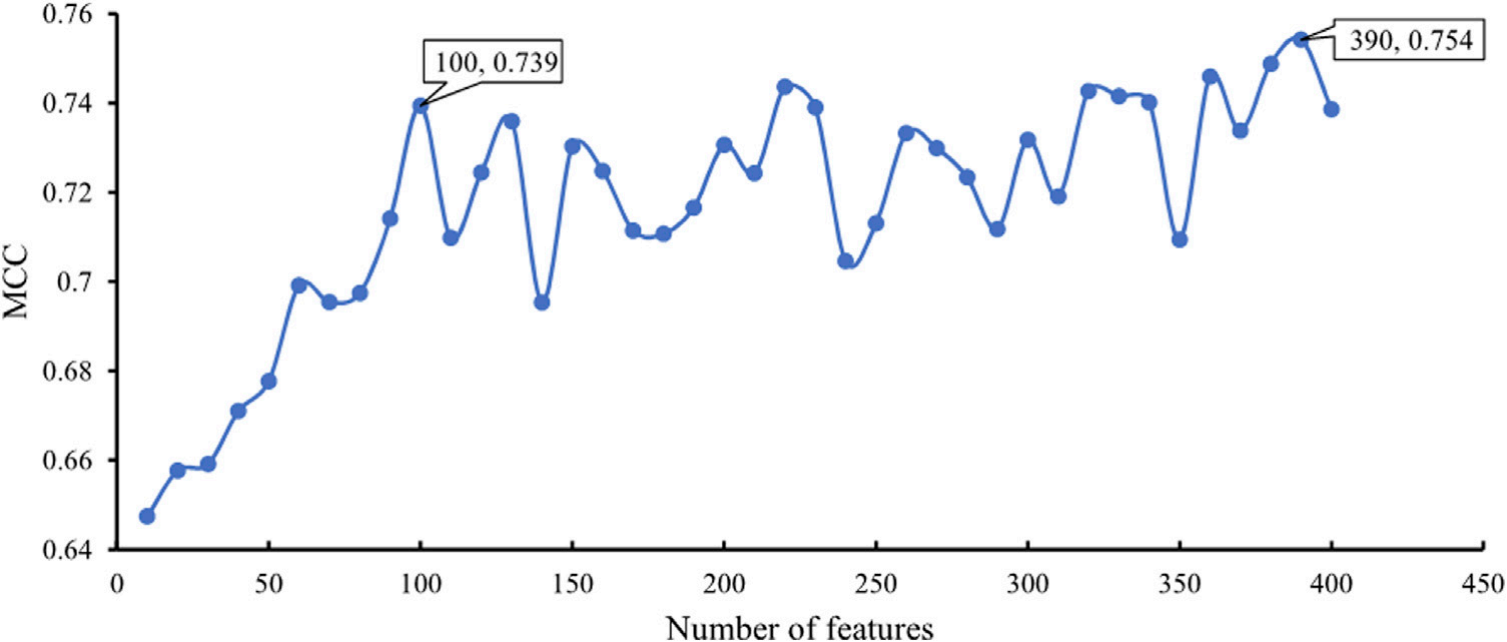
IFS curve with DT classification algorithm on the different number of features. The DT provides the highest MCC of 0.754 when the top 390 features are adopted. DT yields high performance with MCC of 0.739 when only 100 features are used.

In addition to the above SVM algorithm, we used the DT, which is a white-box classification algorithm. In this process, the step size of IFS with the DT was also set to 10, and only the top 400 features in the mRMR list were considered. The IFS results are also available in Supplementary Table S2, and the IFS curve is presented in Figure 4. The best DT classifier yielded an MCC value of 0.754, which was based on the top 390 features. The overall accuracy of this classifier was 0.771, as listed in Table 2. Likewise, we also wanted to obtain an accepted classifier that used few features and provided high performance. As can be seen from Figure 4, the MCC reached 0.739 when the top 100 features were used. The overall accuracy was 0.757 (Table 2). They were only a little lower than those of the best DT classifier. Furthermore, the accuracies on 20 cancer types of these two classifiers were also investigated, as illustrated in Figure 3. Evidently, these two DT classifiers were almost at the same level. Therefore, these top 100 features were considered to build the DT classifier.

### Classification Rules

As mentioned above, the DT classifier with the top 100 features exhibited high performance. Thus, we constructed a DT with these features and all samples. Consequently, we obtained 275 rules, which are presented in Supplementary Table S3. The number of decision rules and criteria used for 20 tumor types are shown in Table 1. Each cancer type was assigned some decision rules. The cancer type “Lung” was assigned most decision rules, whereas ‘Fibroblast’ received least rules. The further analysis of these rules can be found in *Analysis of Decision Rules*.

### GO and KEGG Enrichment Analysis

**FIGURE 5:**
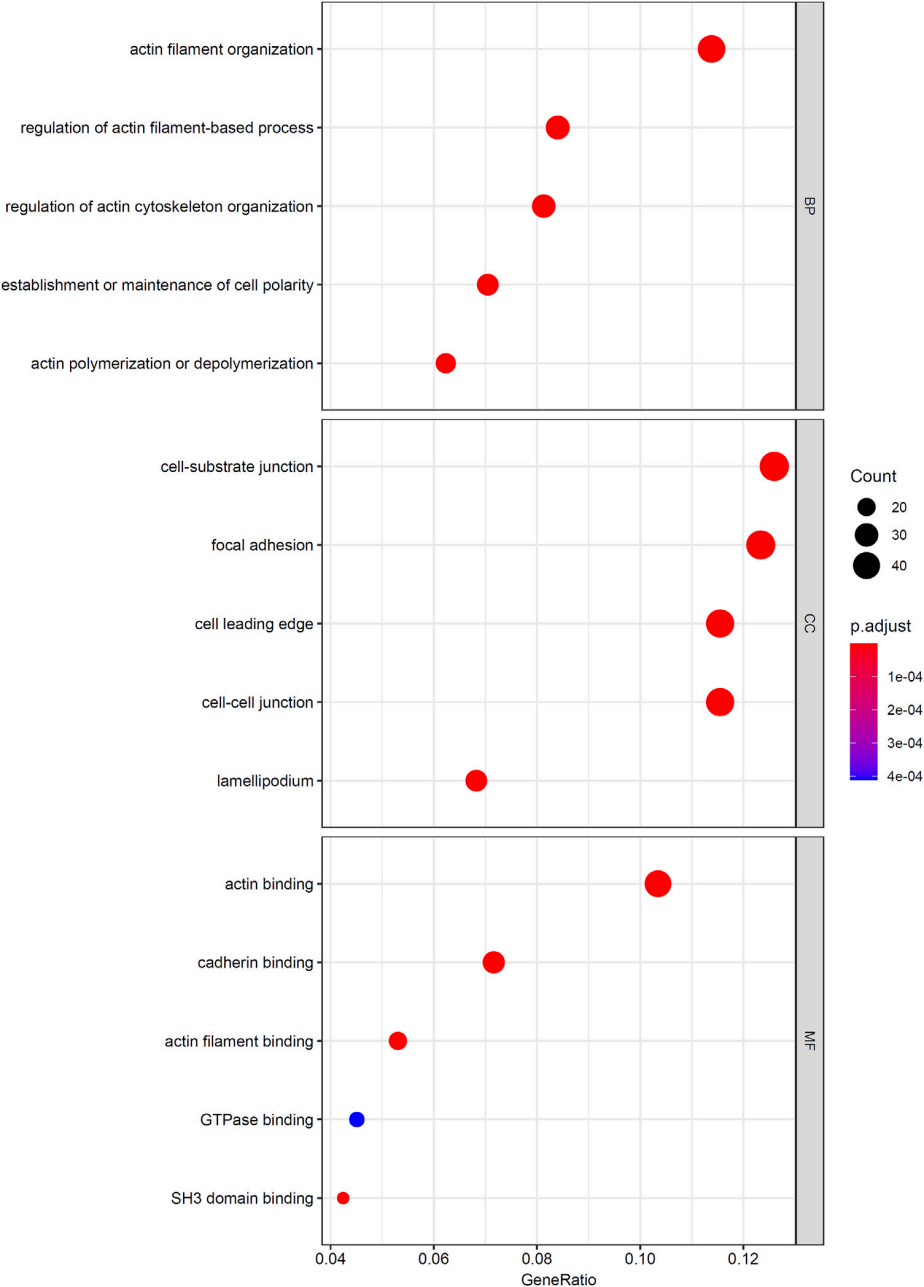
Top enriched GO terms to the top 400 genes in the mRMR feature list.

**FIGURE 6:**
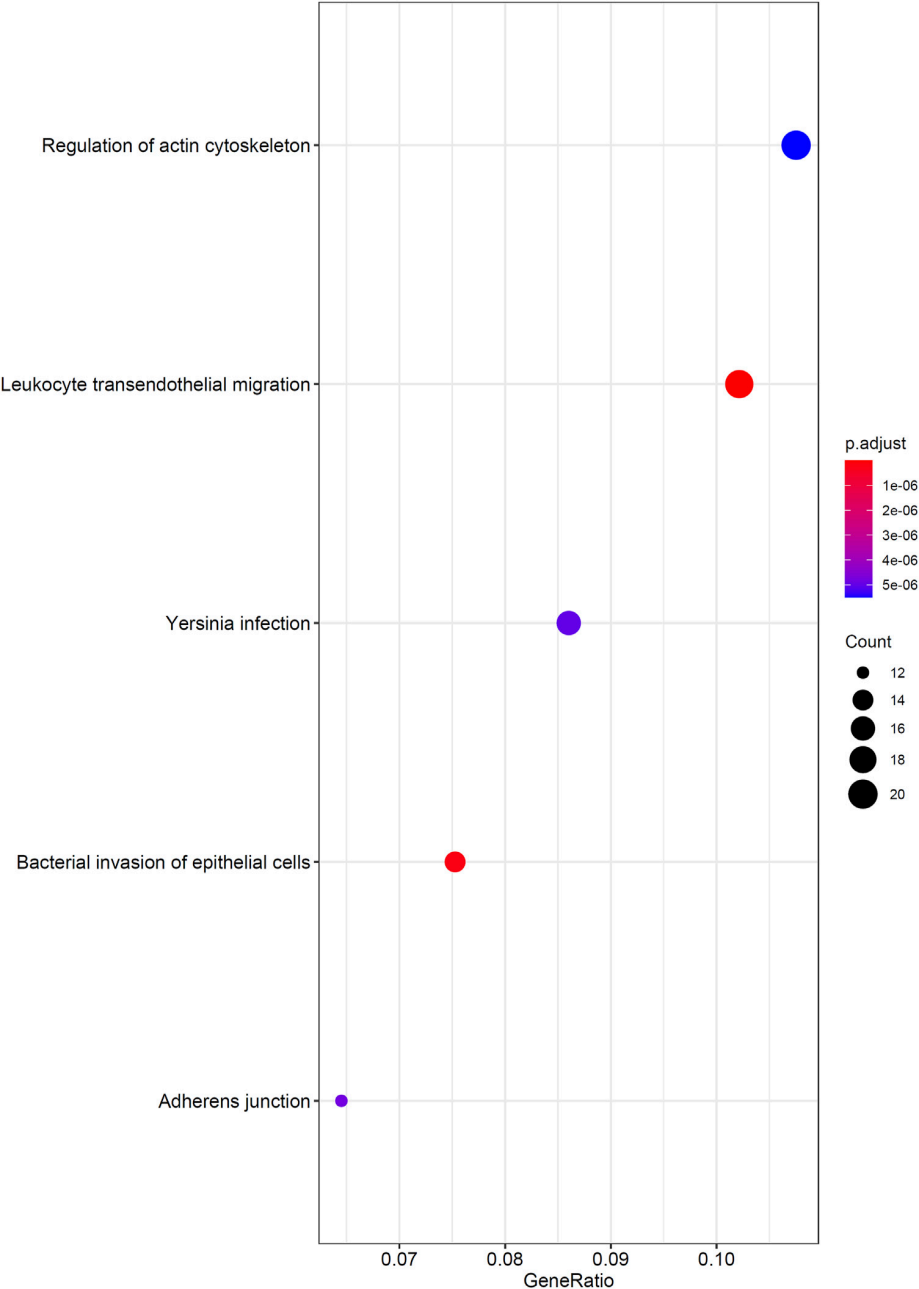
Top enriched KEGG pathways to the top 400 genes in the mRMR feature list.

As mentioned in *Results of the Incremental Feature Selection Method*, the SVM classifiers with top 400 features gave a little lower performance than the best SVM classifier. However, it had much higher efficiency because much less features were used in this classifier. Thus, these 400 features may be highly related to distinguish different cancer types. Thus, we conducted GO and KEGG enrichment analysis on these features (genes). The results can be found in Supplementary Table S4. Some top GO terms and KEGG pathways are illustrated in Figures 5, 6. In *Analysis of Essential Genes*, the discussion on the enrichment analysis results would be given.

## Discussion

In this study, we used the Boruta and mRMR methods to analyze features and applied the IFS method combined with SVM and DT to construct classifiers and decision rules. Some essential features (genes) (see Supplementary Table S1) were extracted. Furthermore, we obtained several decision rules. In this section, we provide an extensive analysis of these essential genes and decision rules.

### Analysis of Essential Genes

Firstly, we performed GO/KEGG enrichment analysis to find whether our 400 selected features were significantly enriched in specific terms. Results were described in *GO and KEGG Enrichment Analysis*, top GO terms and KEGG pathways are illustrated in Figures 5, 6.

We found that the significantly enriched GO terms mainly involve actin organization, cell matrix components and cell polarization. Actin assembly is very important for cell migration, and abnormal regulation of cell migration can drive cancer invasion and metastasis (Yamaguchi and Condeelis, 2007). Different cancers and different differentiation states of cancers often show different patterns of cell migration and the migration of these cancer cells is regulated by various signals. Although it is difficult to use a single strategy to regulate the motility of all cancer cells, inhibiting actin polymerization can inhibit migration of most types of cancer cells (Yamazaki et al., 2005). The loss of cell polarity has been shown to be related to tumor progression (Wodarz and Näthke, 2007). Generally, aggressive tumors lack polarity, and study have shown that different cancers have different abnormal expression or localization of polar proteins, which may also serve as the basis for our classifier (Ellenbroek et al., 2012). The KEGG results also showed similar results, which are mainly related to migration and actin cytoskeleton. This reflects both the importance of cell migration ability to tumors and the difference in invasion of different tumors.

Secondly, among the 400 selected features (genes), the top-ranked genes were usually highly decisive for distinguishing different cell lines. Therefore, some of them were selected for analysis, which are listed in Table 3.

**TABLE 3:** Information of essential genes.

Ensembl ID	Gene symbol	Description
ENSG00000061676	NCKAP1	NCK Associated Protein 1
ENSG00000006327	TNFRSF12A	TNF Receptor Superfamily Member 12A
ENSG00000172037	LAMB2	Laminin Subunit Beta 2
ENSG00000122642	FKBP9	FKBP Prolyl Isomerase 9
ENSG00000070087	PFN2	Profilin 2
ENSG00000141198	TOM1L1	Target Of Myb1 Like 1 Membrane Trafficking Protein

The highest-ranking feature is NCKAP1 (ENSG00000061676). It encodes the NCK-associated protein 1 as a part of the WAVE (WASF) complex that regulates lamellipodia formation. Past studies have revealed that NCKAP1 is associated with multiple types of human cancer. A previous study showed that the WASF3 gene is a promoter of cell invasion in breast cancer and the Nckap1 can keep WASF3 in an inactive conformation through binding to the WASF homology domain at the N-terminus. The activation of WASF3 depends on the combination with RAC1 which can be prevented by the absence of NCKAP1. Thus, the downregulation of NCKAP1 inhibits the activity of WASF3 and may suppresses metastasis in breast cancer cells. In addition, univariate survival analysis have found that high expression level of NCKAP1 is correlated with short overall survival (Teng et al., 2016). The function of NCKAP1 in liver cancer has recently been clarified. Specifically, NCKAP1 can control tumor growth and improve prognosis by enhancing Rb1/p53 activation in hepatocellular carcinoma (HCC) (Zhong et al., 2019). Similarly, a recent study discovered that NCKAP1 is highly expressed in primary non-small-cell lung cancer (NSCLC) and is significantly associated with histologic tumor grade, metastasis, and poor survival rate. It is also related to the HSP90-mediated invasion and metastasis of NSCLC by stimulating MMP9 activation and the epithelial–mesenchymal transition (EMT) (Xiong et al., 2019). In conclusion, NCKAP1 is aberrantly expressed in a variety of cancer types and could be a biomarker and potential therapeutic target.

TNFRSF12A (ENSG00000006327) encodes the receptor of TNFSF12/TWEAK, which is also known as fibroblast growth factor-inducible molecule 14. It can promote endothelial cell proliferation and angiogenesis. Studies have demonstrated that TNFRSF12A is highly expressed in breast cancer, and a high TNFRSF12A level associated with matrix metalloproteinase (MMP)-9 overexpression is related to cancer progression; thus, TNFRSF12A-targeting therapy could improve survival rates in cancer (Yang et al., 2018). Furthermore, through modulating the expression of MMP-9, the overexpression of TNFRSF12A can promote prostate cancer progression and result in poor treatment outcomes (Huang et al., 2011). TNFRSF12A is also highly expressed in human HCC, and *in vivo* experiments have revealed that TNFRS12A knockdown can inhibit cancer cell proliferation and migration (Wang et al., 2017). TNFRSF12A has also been demonstrated to be highly expressed in NSCLC and contribute to NSCLC cell migration and invasion *in vitro* (Whitsett et al., 2012). Other studies have also confirmed that TNFRSF12A is overexpressed in melanomas, gliomas, and esophageal and pancreatic cancers (Han et al., 2005; Tran et al., 2006; Watts et al., 2007; Zhou et al., 2013). Interestingly, in certain tumor types, TNFRSF12A exhibits a low expression level. A study on TCGA data suggested that the downregulation of TNFRSF12A in thyroid cancer could be a potential molecular biomarker for the prediction of poor prognosis (Wu et al., 2020). Therefore, TNFRSF12A has different expression patterns in different cancers and could be a remarkable feature for distinguishing different cancer cell lines. In addition, it could also be a critical therapeutic target, and preclinical studies have shown that the use of inhibitors in cancer with high TNFRSF12A expression has certain effects (Wajant, 2013).

LAMB2 (ENSG00000172037) encodes a subunit of laminins, which are one of the major glycoproteins present in the basement membrane of the extracellular matrix and are related to tumor angiogenesis, invasion, and metastasis. A previous study revealed that the downregulation of LAMB2 caused by HE4 gene interference results in the invasion and metastasis of ovarian cancer cells (Zhuang et al., 2014). Studies on pancreatic cancer have demonstrated that the lack of basement membrane continuity, which is determined by limited laminin expression, is associated with poor postoperative outcomes. In other words, in pancreatic cancer, the downregulation of LAMB2 is correlated with poor prognosis (Van Der Zee et al., 2012).

FKBP9 (ENSG00000122642), which encodes FKBP prolyl isomerase 9, is known to be associated with chaperonin-mediated protein folding and protein metabolism. A recent study has found that FKBP9 could be an independent prognostic marker for predicting the poor prognosis of patients with prostate cancer; that high FKBP9 levels and short biochemical-recurrence-free survival are significantly correlated (*p* = 0.041); and that FKBP9 may be a cancer promoter that enhances prostate cancer progression (Jiang et al., 2020). Another study found that FKBP9 is a critical factor for promoting the malignant behaviors of glioblastoma cells; high FKBP9 level is related to poor prognosis and could confer malignant cells with the capability to resist endoplasmic reticulum stress inducers (Xu et al., 2020). Other studies have also confirmed that FKBP9 is connected with other cancers, such as colorectal and breast cancers (Bianchini et al., 2006; Chang et al., 2020). Thus, FKBP9 may be an effective feature of many cancer cell lines.

PFN2 (ENSG00000070087) encodes an actin monomer-binding protein. It participates in regulating actin aggregation in response to extracellular signals and cell motility. Recently, PFN2 has emerged as a key regulator of cancer development and progression. PFN2 has been reported to be highly expressed in triple-negative breast cancer (TNBC); it could promote the proliferation, migration, and invasion of TNBC cells and may be partially responsible for the worsened survival associated with high PFN2 levels (Ling et al., 2021). In esophageal squamous cell carcinoma, a high PFN2 level is related to short overall survival. Moreover, PFN2 expression is positively associated with tumor invasion depth and lymph node metastasis (Cui et al., 2016). Another study demonstrated that PFN2 is highly expressed in head and neck squamous cell cancer (HNSCC) tissues and cell lines and that the activation of the PI3K/Akt/β-catenin signaling pathway by PFN2 results in the proliferation and metastasis promotion of HNSCC, whereas PFN2 knockdown produces the opposite effects (Zhou et al., 2019). However, another study suggested a different result: the degree of tumor metastasis is negatively associated with PFN2 expression level likely because of the enhancement in EMT induced by low PFN2 levels considering that enhanced EMT may increase migratory capabilities (Zhang H. et al., 2018). Other studies have also found that PFN2 has different expression patterns and effects in NSCLC, small cell lung cancer, and gastric cancer (Hippo et al., 2002; Yan et al., 2017; Cao et al., 2020). In conclusion, PFN2 plays an important role in a variety of cancers and could be an important biomarker for different cancer cells, as well as an attractive therapeutic target.

TOM1L1 (ENSG00000141198) encodes the target of myb1-like 1 membrane trafficking protein. ERBB2-induced breast cancer cell invasion has been documented to be caused by the TOM1L1-derived membrane delivery of MT1-MMP, and ERBB2 and TOM1L1 are frequently coamplified in the breast (Chevalier et al., 2016). Other studies have also found that TOM1L1 is related to colorectal cancer and is highly expressed in bladder cancer (Emaduddin et al., 2008; Zhang Y. et al., 2018).

As analyzed above, the selected genes from our results showed strong expression differences in multiple cancer cells. These genes could be good therapeutic targets. By the same token, distinct gene expression patterns could also be remarkably decisive features for different cancer cell lines.

### Analysis of Decision Rules

Previously, we constructed 275 decision rules on the basis of the top 100 selected features and all cell lines. Each rule contained several criteria. The numbers of rules and criteria for each cancer type are listed in Table 1. In addition, the number of genes involving rules for each cancer type is also listed in this table. In the following, we provide our interpretation and experimental evidence for some rules based on published literature. These evidences indicate the effects of the high/low expression of key genes on tumors which also found to have similar expression patterns in the decision rules of the corresponding tumor cell line (relatively high/low expression level compared to other cell lines).

The 23 rules for identifying breast cancer cell lines included 325 criteria, which involved 62 genes. These genes have considerable experimental support, and here we show some evidence. LDHB (ENSG00000111716) encodes the B subunit of lactate dehydrogenase enzyme, which participates in glycolysis. A study found that LDHB is specifically upregulated in basal-like TNBC, and the loss of LDBH arrests tumor growth *in vivo* (Cui et al., 2015). One study discovered that PAX8 (ENSG00000125618) is the best discriminatory marker between ovarian and breast carcinomas (Nonaka et al., 2008). The same study also reported that PAX8 is negatively expressed in serous carcinoma but is positively expressed in breast carcinomas. This expression pattern is in agreement with our decision rules. RAB34 (ENSG00000109113) regulates the spatial distribution of lysosomes, secretion, and micropinocytosis and is expressed at high levels in breast cancer cell lines. A recent study has found that RAB34 is overexpressed in breast cancer and that the high expression of RAB34 is closely linked to breast cancer cell adhesion, migration, and invasion (Sun et al., 2018).

Among the 275 rules, 51 could identify lung cancer cell lines with 740 criteria involving 80 genes. Here, we provide clear experimental evidence for some genes that are well established in the literature. SOX10 (ENSG00000100146) is a transcription factor that encodes genes involved in the regulation of embryonic development and cell-fate decisions. Studies have demonstrated that SOX10 is usually overexpressed in multiple cancers. It can activate stem/progenitor cells through the Wnt/β-catenin signaling pathway and induces mesenchymal transformation expression (Zhou et al., 2014; Miettinen et al., 2015). However, it appeared in all the lung cancer decision rules with a low expression. A recent experimental study on 1085 NSCLC tumor tissue samples has given direct support for our results. A microarray analysis study revealed that SOX10 is negatively expressed in NSCLC, with only 5 (<1%) cases showing positive results (Kriegsmann et al., 2018). ARHGAP30 (ENSG00000186517) encodes Rho GTPase-activating protein 30, which plays an important role in cell adhesion and cytoskeleton organization regulation. It is downregulated in lung cancer cell lines. Moreover, a low ARHGAP30 level is associated with the activation of Wnt/β-catenin signaling pathways and further leads to lung cancer cell proliferation, migration, and invasion (Mao and Tong, 2018). CTDSPL/RBSP3 (ENSG00000144677) was also downregulated in our rules. It has been reported to be a tumor-suppressor gene in multiple cancers (Kashuba et al., 2009) and to be downregulated in lung cancer (Senchenko et al., 2010). TSPAN4 (ENSG00000214063) was highly expressed in our rules. The transcriptional product of TSPAN4 is a circular RNA that is upregulated in lung adenocarcinoma; circ-TSPAN4 can promote metastasis by increasing the expression of ZEB1 (Ying et al., 2019). In our rules, S100A13 (ENSG00000189171) was required to be relatively highly expressed. As has been seen in another study, S100A13 is overexpressed in NSCLC, especially in the advanced stage. High S100A13 level is strongly associated with tumor angiogenesis and poor prognosis (Miao et al., 2018).

Liver cancer cell lines had nine decision rules containing 115 criteria. These criteria involved 42 genes. The expression patterns of many genes in these rules have been confirmed in several other studies. A1BG-AS1 (ENSG00000268895) is a RNA gene, and its transcriptional product is a lncRNA. A study found that A1BG-AS1 inhibits HCC cell proliferation, migration, and invasion *in vitro*. Clinical association analysis revealed that A1BG-AS1 is downregulated in HCC, and low A1BG-AS1 level is also associated with advanced tumor stage, microvascular invasion, and high tumor grade (Bai et al., 2019). CMTM4 (ENSG00000183723) also showed a low expression level in our rules. As found in other studies, CMTM4 plays a tumor-suppressor role in HCC, wherein it inhibits tumor activities by regulating cell growth and cell cycle (Bei et al., 2017). Thus, consistent with our results, CMTM4 showed negative expression in HCC. AKAP1 (ENSG00000121057) encodes A-kinase anchoring protein 1 and plays an important role in the regulation of mitochondrial function and oxidative metabolism. A previous study identified that AKAP1 is overexpressed in HHC; this expression pattern also provides supporting evidence for our decision rules. AKAP1 may contribute to tumor progression and result in poor overall and disease-free survival rates in patients with HCC (Yu et al., 2018).

The identification of ovarian cancer cell lines had 25 rules with 306 criteria. These criteria involved 67 genes. The validity of our results was supported by other studies that have confirmed some of these genes. As mentioned in the rules for breast cancer cell lines, PAX8 is highly expressed in ovarian cancer and could be a remarkable feature for discriminating between breast cancer and ovarian cancer (Nonaka et al., 2008). In addition, another study found that the knockdown of PAX8 significantly reduces cancer cell proliferation, migration, and invasion (Di Palma et al., 2014). GNAI2 (ENSG00000114353) encodes heterotrimeric G protein, which plays a direct role in regulating the cAMP response element-binding protein. In agreement with our findings, the results of the direct sequencing and qPCR analysis of 589 human ovarian cancer revealed that 85.9% (506) of patients have decreased GNAI2 messaging (Raymond et al., 2014). SRPX (ENSG00000101955) is reported to be a tumor-suppressor gene and is downregulated in multiple cancer cells and tissues (Tambe et al., 2016). This result is consistent with our decision rules for endometrial, pancreatic, and urinary tract cancers. However, one difference is worth noting: we found that SRPX was overexpressed in most rules for ovarian cancer. The overexpression of SRPX has been affirmed by a recent study based on clinical specimens, wherein the upregulation of SRPX is associated with tumor invasion and migration activity in ovarian cancer (Liu et al., 2019).

At the same time, we noted the exclusive genes for some cancer cell line may be quite important. For example, CD276 (ENSG00000103855) only been shown in the rules of lung cancer cell lines. CD276 (B7-H3) encode a member of the immunoglobulin superfamily and is an important immune checkpoint member of the B7/CD28 families. It is induced by antigen presenting cells and participates in the regulation of T cell-mediated immune response (Picarda et al., 2016). Studies have found that CD276 is associated with Mycoplasma pneumoniae pneumonia. It is up-regulated in patients’ plasma and may be involved in the progression of pneumonia by increasing the concentration of TNF-α and the activation of neutrophils (Chen et al., 2013). At the same time, CD276 is also abnormally expressed in a variety of tumors and participates in tumor proliferation, apoptosis, differentiation, invasion and interepithelial transformation. Usually CD276 is up-regulated in tumors and is associated with poor prognosis of patients (Liu S. et al., 2021). In NSCLC, a previous meta-analysis found that the high expression of CD276 was significantly associated with patients’ lymph node metastasis and advanced TNM staging (Wu et al., 2016). Other studies have also found that the expression of CD276 is related to the smoking history and pathological types of patients. Usually, the expression of CD276 in patients with lung adenocarcinoma or smoking history is associated with a shorter overall survival (Inamura et al., 2017; Zhang and Hao, 2019). Although CD276 is highly expressed in a variety of tumors, and its molecular mechanism to promote cancer progression is not clear, our results show that it may be more important for lung cancer. At the same time, other study also found that CD276 was up-regulated in tumor cells of lung cancer patients treated with trametinib, which can achieve better therapeutic effect after combined B7-H3 × T cell bispecific antibody treatment, and this also proves that CD276 is a potential therapeutic target for lung cancer.

We provided pieces of evidence for some decisive genes in the decision rules for four classes of cancer cell lines in the preceding discussion. Although these genes also have different expression patterns in other cancer cell lines and a large number of remaining genes have not yet been explained in detail, we can confirm the reliability of our results from the substantial evidence that we presented. Notably, some distinctive and decisive genes in our rules have not previously been investigated by other researchers. These genes may give new insight into tumor growth and progression, as well as novel potential therapeutic targets.

## Conclusion

This study gave a computational investigation on the cell line gene expression data of cancer cell lines. Several machine learning algorithms were applied on such data. On one hand, we constructed efficient classifiers, which can be latent tools to identify different cancer types. On the other hand, a new set of potential biomarkers (*NCKAP1*, *TNFRSF12A*, *LAMB2*, *FKBP9*, *PFN2*, *TOM1L1*) and expression rules for the identification of different cancers at the transcriptome level were discovered. These biomarkers and rules can be useful materials to uncover mechanism underlying different cancer types, thereby improving our understanding on cancer.

## Data Availability

Publicly available datasets were analyzed in this study. This data can be found here: https://figshare.com/articles/dataset/scRNA-seq_Datasets/7174922?file=14847260.
